# Blind detection of circular image rotation angle based on ensemble transfer regression and fused HOG

**DOI:** 10.3389/fnbot.2022.1037381

**Published:** 2022-12-14

**Authors:** Wenxin Dong, Jianxun Zhang, Yuechuan Zhou, Linfeng Gao, Xinyue Zhang

**Affiliations:** ^1^College of Computer Science and Engineering, Chongqing University of Technology, Chongqing, China; ^2^Sydney Smart Technology College, Northeastern University at Qinhuangdao, Qinhuangdao, China

**Keywords:** image rotation, blind detection, ensemble transfer regression, HOG, loss function

## Abstract

**Introduction:**

Aiming at the problems of low accuracy in estimating the rotation angle after the rotation of circular image data within a wide range (0°–360°) and difficulty in blind detection without a reference image, a method based on ensemble transfer regression network, fused HOG, and Rotate Loss is adopted to solve such problems.

**Methods:**

The proposed Rotate Loss was combined to solve the angle prediction error, especially the huge error when near 0°. Fused HOG was mainly used to extract directional features. Then, the feature learning was conducted by the ensemble transfer regression model combined with the feature extractor and the ensemble regressors to estimate an exact rotation angle. Based on miniImageNet and Minist, we made the circular random rotation dataset Circular-ImageNet and random rotation dataset Rot-Minist, respectively.

**Results:**

Experiments showed that for the proposed evaluation index MSE_Rotate, the best single regressor could be as low as 28.79 on the training set of Circular-ImageNet and 2686.09 on the validation set. For MSE_Rotate, MSE, MAE, and RMSE on the test set were 1,702.4325, 0.0263, 0.0881, and 0.1621, respectively. And under the ensemble transfer regression network, it could continue to decrease by 15%. The mean error rate on Rot-Minist could be just 0.59%, significantly working easier in a wide range than other networks in recent years. Based on the ensemble transfer regression model, we also completed the application of image righting blindly.

## 1. Introduction

In recent years, rotation angle estimation plays quite an important role in many scenarios, such as estimating the rotation angle in radar images (Wang and Jiang, 2008; Zhou et al., 2021b), industrial forgery detection (Hurrah et al., 2021), forensic analysis of digital images (Wei et al., 2010), drone-assisted visual coverage (Cao et al., 2021), etc.

Accurate detection of image rotation angle has irreplaceable important value for industrial production and social life in multiple situations. But how to detect the rotation angle of the image, especially how to blindly detect the rotation angle of a single image, has been a problem for a long time.

In today's Computer Vision field, we can solve such a problem with the help of Transfer Learning. Transfer Learning aims at improving the accurate representation of high-dimensional and sparse data on target domains by transferring the knowledge contained in different but related source domains (Zhuang et al., 2021; Wu et al., 2022). In this way, the dependence on a large number of target-domain data can be reduced for constructing target learners. Due to the wide application prospects, Transfer Learning has become a popular and promising area in Deep Learning.

Transfer Learning can solve classification and regression tasks very well. For the task of rotation angle estimation, such as “image righting,” it is more appropriate to regard it as a regression task, because the goal of regression is to predict specific values. If we regard it as a classification task, the specific values can be accurately predicted unless it is divided into 361 categories (0–360°, divided into 1 category according to each 1°) or more categories. But this puts forward higher requirements for the magnitude of the data, which is not easy to solve.

Previously, many scholars have studied the task of estimating the rotation angle of the image, but their researches were rare for full circular data, which will be a new challenge. At the same time, the range of rotation angle studied by many scholars is relatively limited, such as limited to 45° (Wei et al., 2010), or limited to a rough range (Zhou et al., 2019). For the range of the rotation angle expanded to 0–360°, how to estimate it accurately is also a big problem. Some strategies required the help of a reference image when estimating the rotation angle (Onishi and Suzuki, 1996; Kim and Kim, 1999; Xiong and Quek, 2006; Revaud et al., 2009). However, in many cases, we do not have the original image for reference, so how to perform blind detection of rotation angle without a reference image is also one of our goals to be solved. What we call “blind” is that the image before rotation is unknown, and the only available data is a single image (Goljan, 2018; Zhou et al., 2018).

Aiming at the processing of circular images, the study of large-scale rotation angle, and the obtaining of relatively accurate blind estimation of the rotation angle, this paper proposed the ensemble transfer regression network (ETRNet). The histogram of oriented gradients (HOG) was used to extract the subtle directional features of the circular image. Combined with the newly Rotate Loss, a more relatively accurate blind estimation of the image rotation angle could be reached.

Our main contributions include:

**(1)** The ensemble transfer regression network, capable of blind detection of rotation angles in circular images, was proposed by voting with multiple best regressors.

**(2)** HOG-based directional feature fusion strategy was proposed. Feature learning was carried out with the fused HOG feature image, which added additional directional information to the original image.

**(3)** Rotate Loss proposed to solve the wrong judgment of degree difference between the predicted angle and the reference angle. The degree difference could be too large near 0° when the rotation range is expanded to 0–360°.

**(4)** The task of blindly righting circular random rotation images has been generally solved.

The format of this paper is as follows: Section 2 discusses existing approaches and related work. In Section 3, we present the overall architecture of ETRNet. The directional feature of the image is extracted based on HOG, and a new rotation angle loss function Rotate Loss is proposed. Section 4 introduces the production process of the circular dataset and the design ideas of the regressor. The validity of Rotate Loss and the effect of fused HOG were verified. An application of image righting blindly was also completed. In Section 5, some experimental results in Section 4 are analyzed and discussed. The final section makes a summary and puts forward the direction of future efforts.

## 2. Related work

In the research on the detection method of image rotation angle, many excellent methods have emerged. It can be roughly divided into non-blind detection methods or blind detection methods.

### 2.1. Non-blind detection method of image rotation angle

In Onishi and Suzuki (1996) applied a modified version of the Hough transform to the reference and input images, and computed the angle of rotation uniquely. In 1999, a robust method of estimating a rotation angle using the phase information of Zernike moments was presented by comparing two graphs (Kim and Kim, 1999). In Xiong and Quek (2006) created an angle histogram with a voting procedure. The rotation angle between the reference image and the observation image could be determined by seeking the orientation difference that corresponded to the maximum peak in the histogram. In Revaud et al. (2009) retained the phase information, improved the Zernike moment, and retrieved the rotating image of the randomly rotated image under the conditions of adding noise, deformation, occlusion, and translation. In the case of noise and deformation, they could achieve the accuracy of the average root mean square error (RMSE) within 1°.

### 2.2. Blind detection method of image rotation angle

In Fukumi et al. (1997) relied on neural networks to roughly estimate the rotation angle of numbers and coins. In Ulas et al. (2007) proposed a method based on parameter statistics. Using 1D and 2D linear models and statistical parameters of the X-axis, Y-axis, and diagonal axis, they proposed a fabric rotation angle estimation method. When the image resolution was high enough, the error within plus or minus 1° can be obtained between –30 and 30°. In Wang and Jiang (2008) proposed a rotation angle estimation method of ISAR imaging, which estimated the optimal rotation rate according to the received signal to estimate the rotation angle.

In Wei et al. (2010) developed an image rotation angle estimator based on the relations between the rotation angle and the frequencies at which peaks due to interpolation occured in the spectrum of the image's edge map. In Qian et al. (2013) proposed a blind image rotation angle estimation method by exploring the periodicity of pixel variance of rotated images. Experiment results showed that their method worked well for rotation angles larger than 5°. In Chen et al. (2014) used the two-dimensional spectrum of image second-order statistics and used the hidden periodicity in the rotating image to estimate the rotation angle. The normalized range was improved but the prediction range was within one quadrant.

In Deng et al. (2018) designed a derotation layer, which explicitly rotated a feature map up to a given angle. For rotation angles >30°, there was an 80% prediction rate. Their work well demonstrated the ability of a deep regression network to predict rotation angles. The same year, Goljan (2018) utilized the Linear Pattern (LP) as a global template. In particular, no side information, such as a watermark or the EXIF header, was required. Their method was generally applicable whenever the image under investigation had a strong LP before rotation. The main advantage of their proposed method was its accuracy in estimating small rotation angles (< 3°). It could also work after resizing.

In 2019, directional wavelet and horizontal wavelet were used for preprocessing (Rodriguez et al., 2019). Then it passed through a network with a pooling layer, convolution layer, and dense layer. Finally, a two-dimensional vector was output as the result. The maximum value of the column was taken as the classification result, and the maximum value of the row was taken as the angle prediction. The prediction error rate on MNIST-R was 2.69%. Same in Zhou et al. (2019) used the shifting pixels method and the octagonal convolutional kernel to construct the angle prediction network. However, for angles that were not equal to n × 45° (*n* = 1, 2, ..., 7), the network could only estimate the angle roughly for the reason that they only chose 45° as the stride to rotate the parameters in convolutional layers.

In 2021, the approach of Hurrah et al. (2021) was based on the premise that the rotation of an image resulted in the formation of uniform intensity patches. After detecting sharp boundaries through horizontal/vertical scanning, the angle of rotation was estimated blindly without the need for a reference image. An angle was estimated accurately for a range of 1° to ±89°. Same in 2021, DTCWT, which had good directional selectivity, was used instead of DWT to extract rotated LP (Zeng et al., 2021). Once the noise residuals were extracted, a coarse-to-fine strategy was used to search for possible rotation angles.

From the above research, some findings are as follows:

**(1)** The blind detection method of image rotation angle is more challenging than the non-blind method, and it is also a hot spot of recent research. However, the blind detection method focuses on the specific strategies that can be adopted in specific tasks. For example, the strategy adopted in Hurrah et al. (2021) relied on the patches generated by the rotation of square images to further predict the rotation angle by detecting the rotation boundary.

**(2)** Their strategies were inapplicable for circular images, because circular images could not produce special triangular patches after rotation, and might not rely on detecting rotation boundaries to predict rotation angles.

**(3)** The rotation angle range of some studies was also limited. For example, the estimation range of Hurrah et al. (2021) was 1° to ±89°.

**(4)** Others could only find an approximate range, but can not tell a more accurate angle. It was clear that Zhou et al. (2019) needed to use a smaller rotation step to make a more accurate prediction.

It can be seen that the processing of circular images, the study of large-scale rotation angles, and the obtaining of relatively accurate prediction of rotation angles are currently urgent problems to be solved. These three problems form the focus of this paper.

## 3. The overall architecture

**Figure 1:**
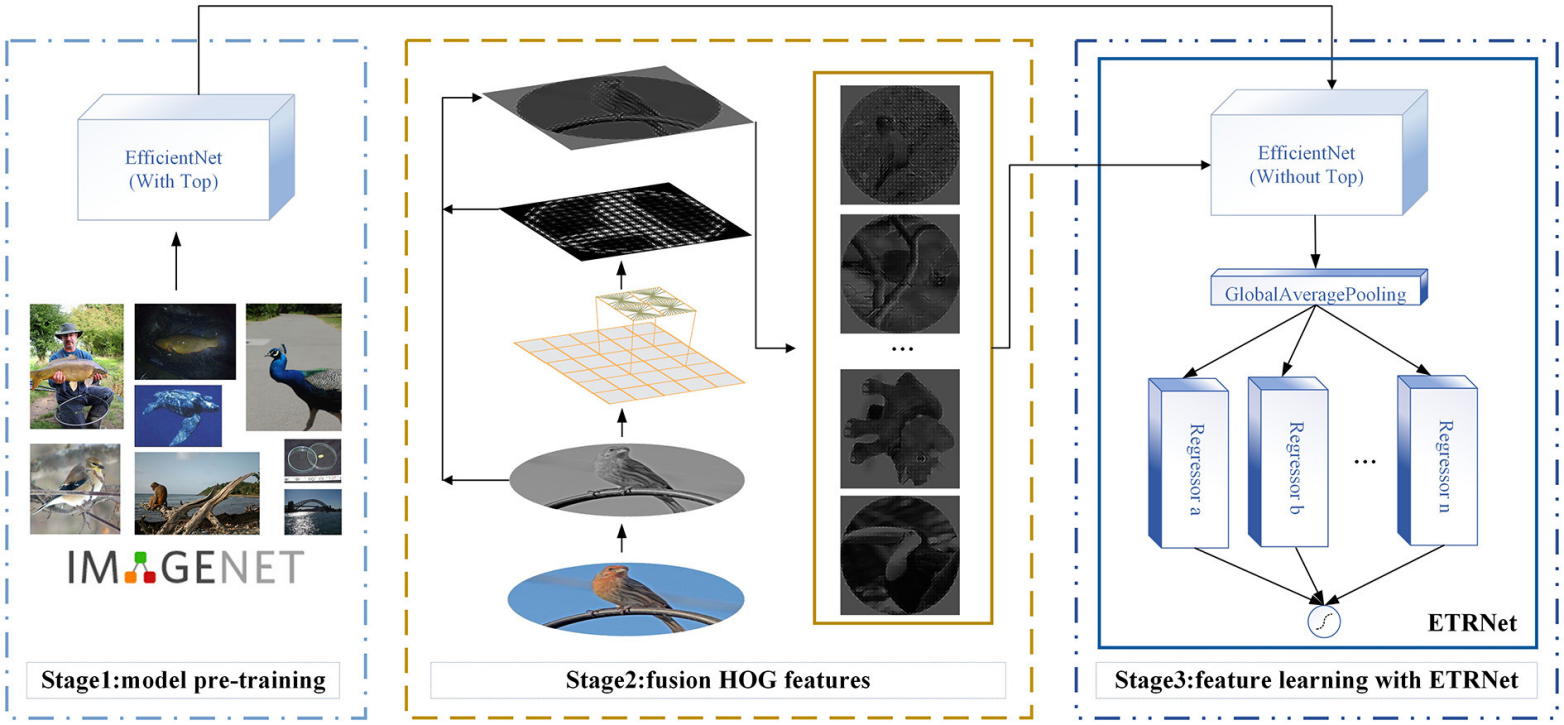
Transfer learning regression network model.

In this paper, we proposed the HOG (Dalal and Triggs, 2005) based directional feature fusion strategy, and on this basis, combined with the ensemble transfer regression network (ETRNet) to blindly predict the rotation angle. At the same time, our proposed Rotate Loss was used to make the model have better convergence. The overall architecture can be shown in Figure 1.

### 3.1. HOG-based directional feature fusion strategy

At the 2005 CVPR conference, French researchers Dalal and Triggs proposed the HOG, which could be a feature descriptor used in image processing for object detection. At that time, the combination of HOG operator and SVM classifier in static image pedestrian detection achieved certain results and began to be widely used.

Fusion methods could be used for identifying salient regions (Tian et al., 2021; Zhou et al., 2021a). HOG can extract more obvious directional features, but HOG alone is not enough to complete the subsequent rotation angle prediction task. Because the feature map obtained by HOG only retains directional information and loses the original semantic information of the image about vision, object, concept, etc.

**Figure 2:**
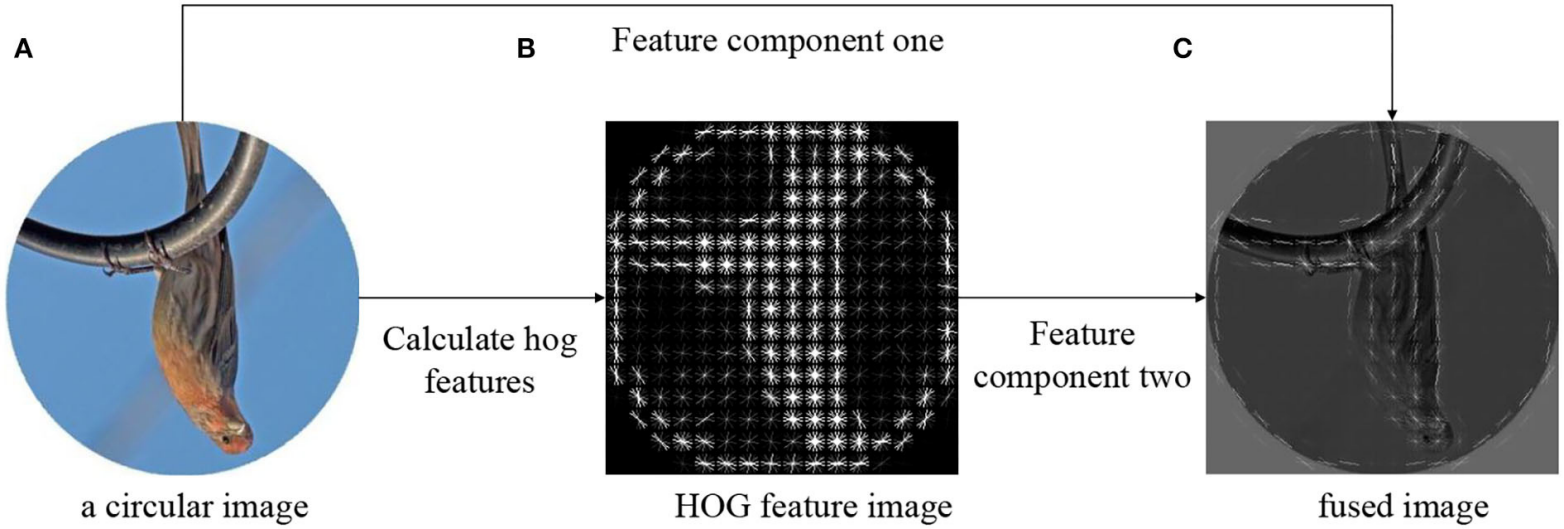
The fusion process of HOG directional feature and original image. **(A)** The original image. **(B)** The directional feature of HOG. **(C)** The fusion effect according to Equation (1).

Therefore, the strategy of fusing HOG features and original image features was designed according to (1), and the fusion effect can be shown in Figure 2.


(1) $$\begin{array}{l} I_{new}\left(x,y\right)=addWeighted\left(\alpha \cdot H\left(x,y\right),\beta \cdot I\left(x,y\right)\right) \end{array}$$

where *addWeighted* means adding according to different weights, *H*(*x, y*) represents the pixels of the HOG feature map, *I*_*new*_(*x, y*) represents the pixels of the fusion feature, α, β ∈ [0, 1] and α + β = 1.

### 3.2. Ensemble transfer regression network

Transfer Learning is more suitable for the case of limited labeled data (Wu et al., 2021; Zheng et al., 2021; Zhou et al., 2022). The annotated rotating images we got were limited, so we chose the CNN model which was pre-trained on the ImageNet database to make up for the lack of annotated data based on its powerful distinguishing ability. This paper selected EfficientNet (Tan and Le, 2019) as the Transfer Learning base model. EfficientNet, as an efficient and robust convolutional neural network model with good generalization, has achieved good results on the ImageNet dataset and has been widely used as a feature extractor for transfer learning models in recent years (Hoang and Jo, 2021).

The overall network structure is shown in Figure 1. Firstly, the overall parameters of the EfficientNet network were trained by well annotated ImageNet dataset, where the parameters included the weights of the feature extractor and classifier (called TOP). Secondly, in the fine-tuning stage, the parameters of the feature extractor obtained in the first step were fixed. Then the weights of the redesigned TOP were calculated by limited labeled rotating images and updated to obtain a new set of parameters suitable for small sample regression tasks. Thirdly, multiple regressors were combined to form the ensemble transfer regression model. The combination was generated based on the better-performing regressor voting.

The basic parts of the new TOP included a Global Average Pooling layer and a Dense layer with a single neuron activated by Sigmoid as output. The Global Average Pooling layer connected the feature extractor to realize dimension reduction and global feature extraction.

### 3.3. Rotate loss

The loss function can estimate the distance between the prediction and the reference label. The smaller the value of the loss function, the better the prediction effect of the model. The selection of the loss function needs to be based on the specific network and the problems to be solved. Since our goal is to get a more exact prediction of image rotation angle, a more realistic regression loss function needs to be considered.

Mean square error (MSE) is one of the regression loss functions, which is the average of the square distance between the estimated value and the reference value, as shown in Equation (2):


(2) $$\begin{array}{l} MSE=\frac{1}{n}\overset{n}{\underset{i=1}{\sum }}{\left(y_{i}-{\hat{y}}_{i}\right)}^{2} \end{array}$$

where *y* is the actual output of the network, $\hat{y}$ is the expected output (i.e. reference label). However, if there are some particularly unreasonable outliers, MSE will give an exaggerated average, thereby reducing the overall performance of the network model.

Mean absolute error (MAE), as shown in Equation (3), is another commonly used regression loss function to measure the average distance between *y* and $\hat{y}$. Compared with MSE, MAE is more inclusive of outliers. However, due to the existence of absolute values, MAE is not conducive to function convergence and model training.


(3) $$\begin{array}{l} MAE=\frac{1}{n}\overset{n}{\underset{i=1}{\sum }}|y_{i}-{\hat{y}}_{i}| \end{array}$$

Huber loss (Meyer, 2021), as another regression loss function, as shown in Equation (4), combines the advantages of MSE and MAE, and has a strong anti-interference ability for outliers. When the difference between the predicted and the reference is less than δ, it is regarded as a small error. Otherwise, it is regarded as an outlier with a large error and corrected by the second formula in Equation (4).


(4) $$\begin{array}{l} Huber\left(y_{i},{\hat{y}}_{i}\right)=\{\begin{array}{c} \frac{1}{2}{\left(y_{i}-{\hat{y}}_{i}\right)}^{2}\;,for|y_{i}-{\hat{y}}_{i}|\leq \delta \\ \delta \cdot \left(|y_{i}-{\hat{y}}_{i}|-\frac{1}{2}\delta \right)\;,otherwise\; \end{array} \end{array}$$

where, δ is a parameter, usually 0.1 or 1.

But for the rotation angle blind detection task, only relying on Equation (4) is still unreasonable. The optional range of image rotation is 0° to 360°. Therefore, when the rotation angle is near 0°, the Huber loss function cannot be accurately judged. When the predicted value is less than the reference value, for example, the predicted value is 4°, the reference value is 355°, and the actual degree difference is 9° (after normalization, it is 9/360=0.025, far less than δ). In the Huber loss function, the degree difference will be 351°, which will be regarded as an abnormal situation for error processing.

Therefore, based on Equation (4), the improved loss function Rotate Loss, shown as Equation (5), was proposed. It will solve the problem of the wrong judgment of degree difference when the predicted and the reference value are near 0°.


(5) $$\begin{array}{l} R\left(y_{i},{\hat{y}}_{i}\right)=\{\begin{array}{c} \frac{1}{2}{\left(y_{i}-{\hat{y}}_{i}\right)}^{2}\;,for|y_{i}-{\hat{y}}_{i}|\leq \delta_{1}\\ \delta \cdot \left(|y_{i}-{\hat{y}}_{i}|-\frac{1}{2}\delta \right)\;,for|y_{i}-{\hat{y}}_{i}|\leq \delta_{2}\\ \frac{1}{2}{\left[\left(1-{\hat{y}}_{i}\right)+y_{i}\right]}^{2}\;,for\;y_{i}<{\hat{y}}_{i},|y_{i}-{\hat{y}}_{i}|>\delta_{2}\\ \frac{1}{2}{\left[\left({\hat{y}}_{i}+1\right)-y_{i}\right]}^{2}\;,for\;y_{i}>{\hat{y}}_{i},|y_{i}-{\hat{y}}_{i}|>\delta_{2} \end{array} \end{array}$$

The range before normalization is an integer of [0,360], and after normalization is a decimal of [0,1]. δ_1_ is taken as the normalized value of 340, namely 340/360 ≈ 0.944, and δ_2_ is taken as the normalized value of 350, namely 350/360≈ 0.9722.

Rotate Loss mainly added two judgment situations when the predicted and the reference are near 0° and the degree difference is too extreme. When the predicted value is less than the reference, the angle error is corrected to $\left|\left(1-{\hat{y}}_{i}\right)+y_{i}\right|$. Otherwise, the angle error is corrected to $\left|\left({\hat{y}}_{i}+1\right)-y_{i}\right|$.

**Figure 3:**
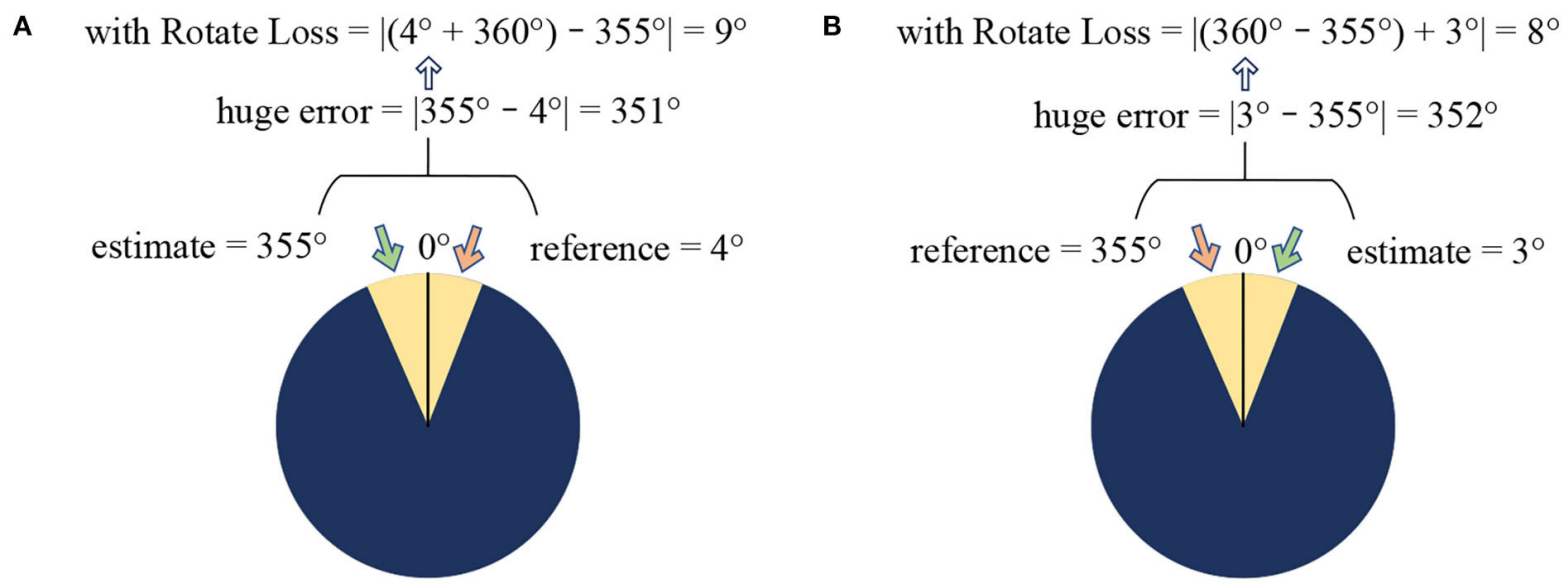
Examples of Rotate Loss. **(A)** Get true error when the estimate bigger than the reference. **(B)** Get true error when the reference bigger than the estimate.

At this time, if the predicted value is less than the reference, it will be considered again. If the prediction is 3° and the reference is 355°, it can be corrected to 8° according to Rotate Loss as shown in Figure 3.

### 3.4. Composition of regressor

The final effect of transfer learning for rotation angle prediction depends not only on the new sample data but also on the design of regressors in ETRNet. To improve the accuracy and generalization of the model, many strategies can be considered, such as whether to add a BN layer, Regularization layer, or Dropout layer.

A) BN

The BN algorithm can be referenced from Ioffe and Szegedy (2015). The architecture with Batch Normalization allows a higher learning rate, so it can generate better benefits in the model with better generalization ability (Simon et al., 2016).

B) Regularization

Regularization can promote the sparsity of deep learning networks, and eliminate redundant connections and unnecessary neurons. In practice, we often consider L1 regularization (Kamalov and Leung, 2020) or L2 regularization (Shi et al., 2019).

C) Dropout Algorithm

By randomly discarding units during training, the network is prevented from overfitting (Baldi and Sadowski, 2014). Dropout can be regarded as a model fusion method.

The above strategies can be used separately or in combination. However, the order of the combination may have an impact on the prediction results. For example, some analysts say that the joint effect of BN and dropout will play a negative role instead (Li et al., 2019). Especially when dropout is applied before BN, it may eventually lead to a worse prediction.

## 4. Experiments

To detect and analyze the image rotation angle more accurately, miniImageNet and Minist are selected as the original data. Based on them, a more challenging circular random rotation dataset Circular-ImageNet, and a random rotation dataset Rot-Minist were produced. A series of comparative experiments were conducted.

### 4.1. Dataset

MiniImageNet is a dataset containing 60,000 colorful images coming from 100 classes, with 600 images in each class (Xue et al., 2020). However, 60,000 images are redundant and cause too much pressure on the equipment. Therefore, we randomly selected 6,000 images as the initial data for further processing. In our experiments, to simulate better challenging circular images, we sequentially processed the initial data as follows:

a) Making a maximum inscribed circle based on the image center.

b) Scaling the image to 320 × 320 pixels.

c) The image is rotated randomly from 0° to 360°(integers) counterclockwise and used as a reference label.

d) HOG features are extracted and fused according to Equation (1) to obtain a fused image.

**Figure 4:**
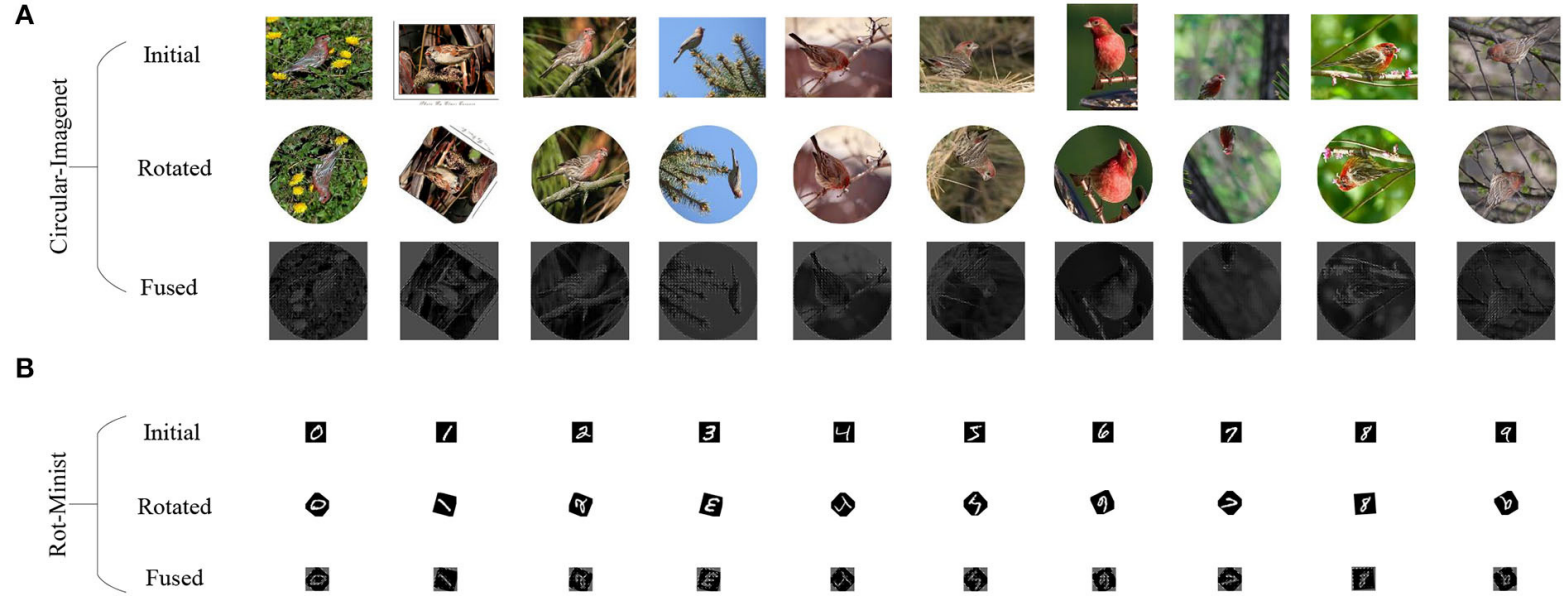
The emergence of the dataset. **(A)** Circular-ImageNet. **(B)** Rot-Minist.

All the above operations are performed on the initial data once to form the data set Circular-ImageNet, as shown in the upper half of Figure 4.

Since the overall quality of the Circular-ImageNet image is relatively clear, image enhancement is not necessary. In addition, we should note that some common data enhancement methods are not suitable for Circular-ImageNet. Such as translation, flip, or rotation. Because these operations will change the image rotation angle information, they can not be directly and accurately consistent with the reference label. For Circular-ImageNet, the total number is 6,000. In this paper, the division ratio of the training, validation, and test sets is 6:2:2.

To compare with other strategies, the Rot-Minist was obtained by 0°–360° random rotation based on Minist, shown as the bottom half in Figure 4. Also divided in a ratio of 6:2:2.

### 4.2. Evaluation indicators

In the regression task, the commonly used evaluation indexes are MSE, MAE, RMSE, etc. MSE and MAE shows as Equations (2) and (3), and RMSE shows as Equation (6).


(6) $$\begin{array}{l} RMSE=\sqrt{\frac{1}{n}\overset{n}{\underset{i=1}{\sum }}{\left(y_{i}-{\hat{y}}_{i}\right)}^{2}} \end{array}$$

The closer MSE, MAE, and RMSE are to 0, the closer the predicted angle value is to the label.

During training and verifying, MSE_Rotate was the mainly used evaluation indicator, as shown in Equation (7). During testing, we observed the feedback of the four indicators: MSE_Rotate, MSE, MAE, and RMSE.


(7) $$\begin{array}{l} MSE\text{\_}Rotate=\frac{1}{n}\overset{n}{\underset{i=1}{\sum }}{\left(360\times y_{i}-360\times {\hat{y}}_{i}\right)}^{2} \end{array}$$

### 4.3. Comparative experiment of loss function

Taking Circular-ImageNet as the data set, the first set of experiments compared the comprehensive performance of the proposed loss function and other loss functions to verify the effectiveness of the proposed Rotate Loss.

**Figure 5:**
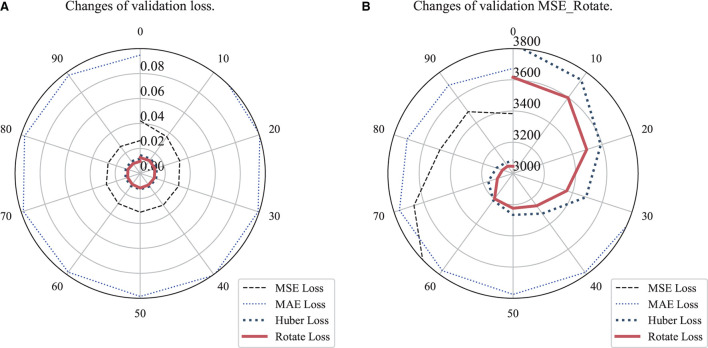
Comparison of loss and MSE_Rotate values among loss functions. **(A)** Changes of loss values. **(B)** Changes of MSE_Rotate values.

MSE, MAE, Huber, and Rotate Loss were respectively added in comparative experiments. EfficientNetB3 with a single regressor, global average pooling with BN, was used here. The result indicated that Rotate Loss performed best both in loss and MSE_Rotate values, as shown in Figure 5.

To achieve a fair comparison between algorithms and prevent the network from reaching the error threshold and ending the training in advance, the minimum value of the loss function was set to 0 and the updated minibatch was 64. The number of epochs was set to 100 to better observe the change among different loss functions. The optimizer used was Adam and the equipment used was NVIDIA RTX A6000. Those experiments were mainly carried out based on Tensorflow and OpenCV.

### 4.4. Comparative experiment of regressor

Experiments comparing the detection accuracy among multiple regressors can tell which one had better performance, as shown in Table 1. ETRNet will be formed by better regressors with better robustness generalization and accuracy. Baseline means no selection.

**Table 1:** Regressor design strategies.

**Regressor**	**BN**	**L1**	**L2**	**Dropout**
Ra	√			
Rb		√		
Rc			√	
Rd				√
Re	√	√		
Rf	√		√	
Rg	√			√
Rh		√	√	
Ri		√		√
Rj			√	√
Rk	√	√	√	
Rl	√	√		√
Rm		√	√	√
Rn	√	√	√	√
Baseline				

**Figure 6:**
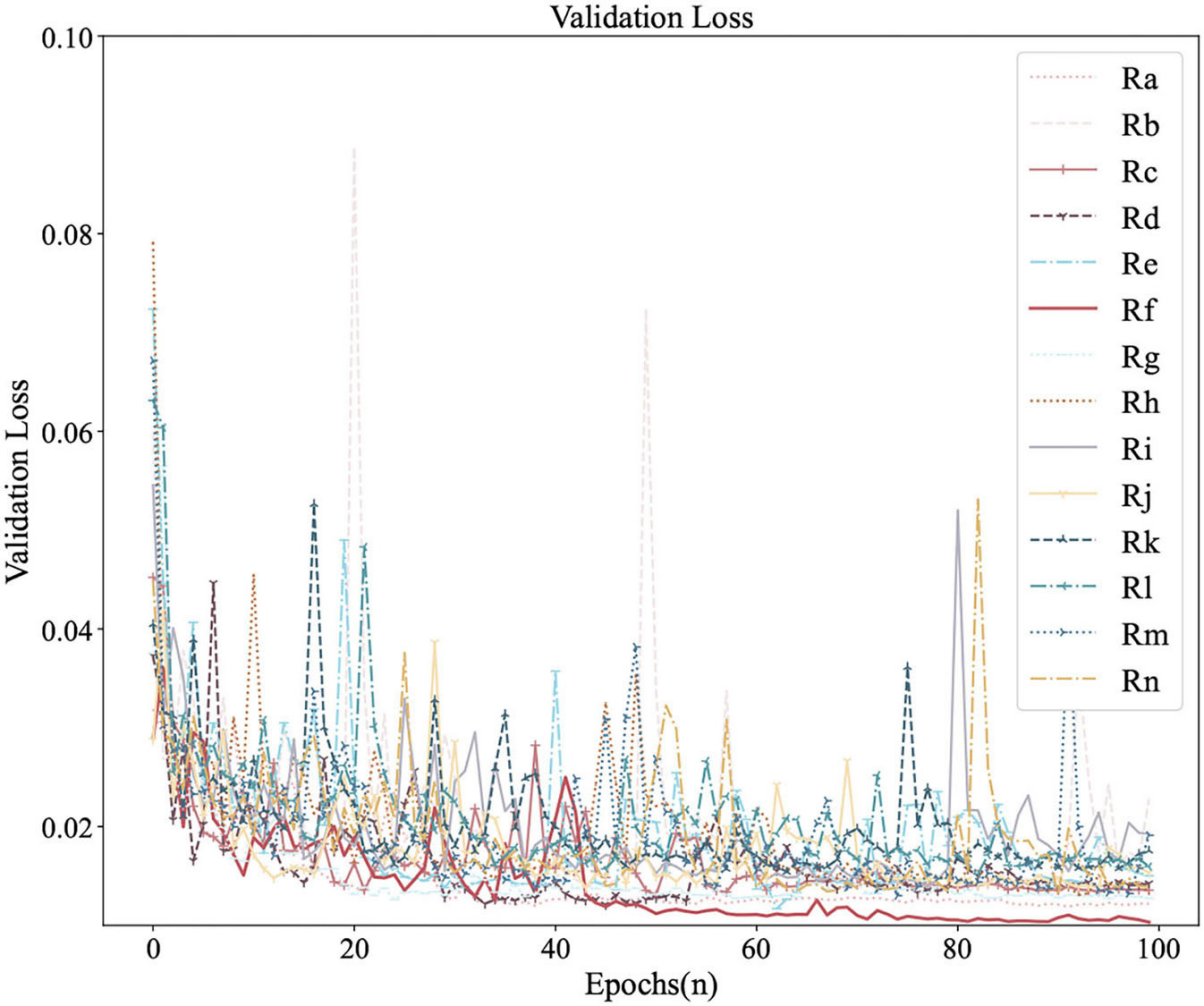
Comparison of Rotate Loss among 14 regressors on the validation.

**Figure 7:**
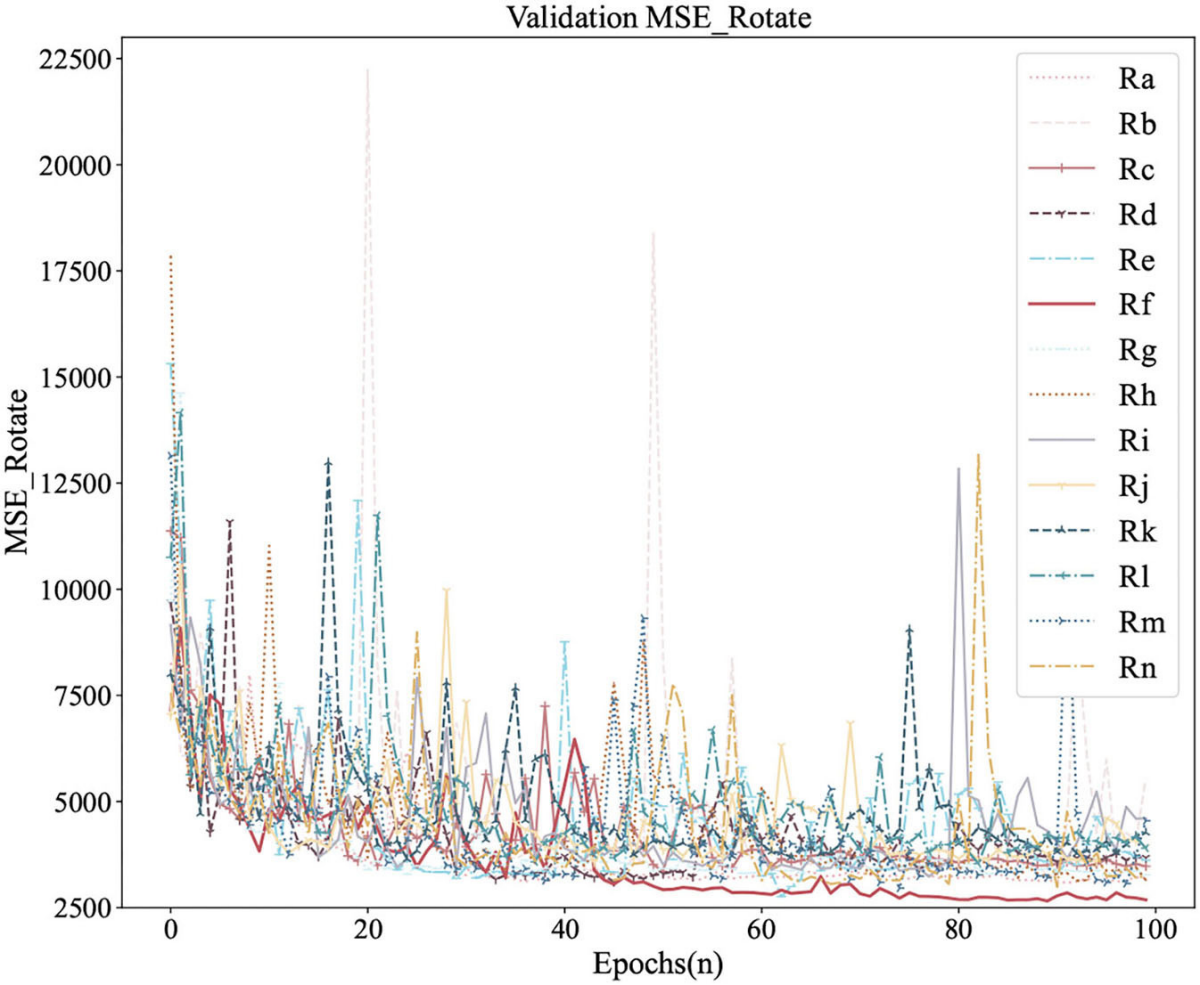
Comparison of MSE_Rotate among 14 regressors on the validation.

Figures 6, 7 were the comparison diagrams of 14 regressors after 100 epochs on the validation set of Circular-Imagenet. Table 2 showed the feedback of MSE_Rotate, MSE, MAE, and RMSE on the test set. The result indicated Re, Rf, Rm, and Rn were significantly better than baseline. At the same time, ETRNet voted based on the four regressions (Re, Rf, Rm, and Rn), and obtained the best effect.

**Table 2:** Comparison of MSE_Rotate, MSE, MAE, RMSE on testset.

**Regressor**	**MSE_Rotate**	**MSE**	**MAE**	**RMSE**
Ra	2,070.5363	0.0320	0.0886	0.1788
Rb	1,993.4750	0.0308	0.0837	0.1754
Rc	2,051.8608	0.0317	0.0932	0.1779
Rd	1,959.2438	0.0303	0.0910	0.1739
Re	**1,702.4325**	**0.0263**	0.0881	**0.1621**
Rf	**1,760.0450**	**0.0272**	0.0808	**0.1648**
Rg	1,962.2700	0.0303	0.0888	0.1740
Rh	1,955.1758	0.0302	0.0896	0.1737
Ri	2,055.9233	0.0317	0.0912	0.1781
Rj	1,957.4579	0.0302	0.0923	0.1738
Rk	2,059.7575	0.0318	0.0970	0.1782
Rl	1,947.0567	0.0301	0.0893	0.1734
Rm	**1,749.5342**	**0.0270**	0.0874	**0.1643**
Rn	**1,768.6558**	0.0273	0.0860	**0.1652**
ETRNet	**1,444.9354**	**0.0223**	**0.0792**	**0.1494**
Baseline	1,770.3567	0.0273	0.0807	0.1653

The bolded part means better than baseline.

### 4.5. Ablation experiment

To verify the effectiveness of HOG, an ablation experiment was carried out. We adopted the best regressor in 4.4 and continued to use EfficientNet as the feature extractor. Rotate Loss was the loss function.

Three groups of comparative experiments were conducted. One was the Circular-ImageNet with only HOG features for training. The second was without the HOG feature. The third one was Rf with fused HOG.

**Figure 8:**
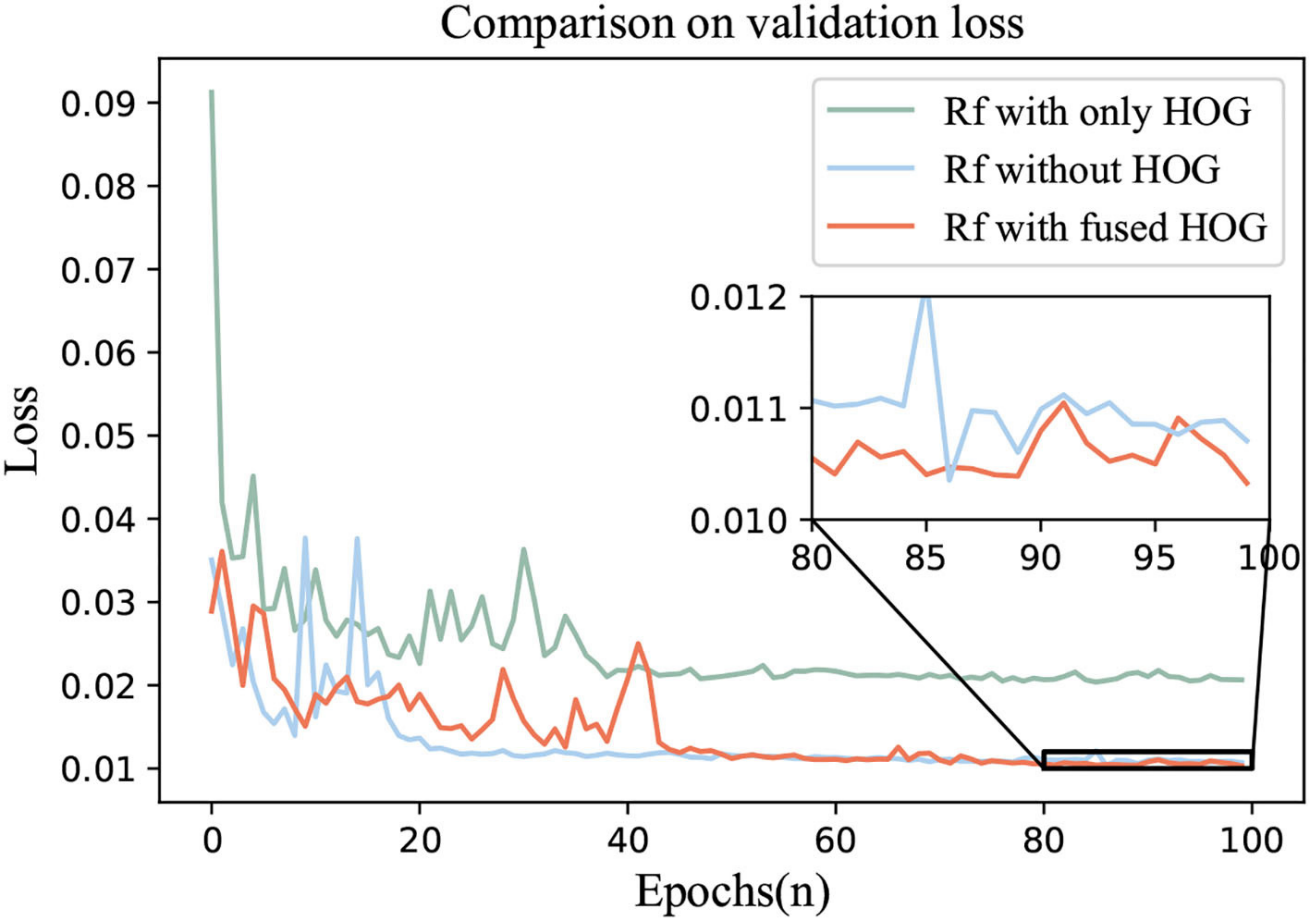
Comparison of loss values in ablation experiment.

**Figure 9:**
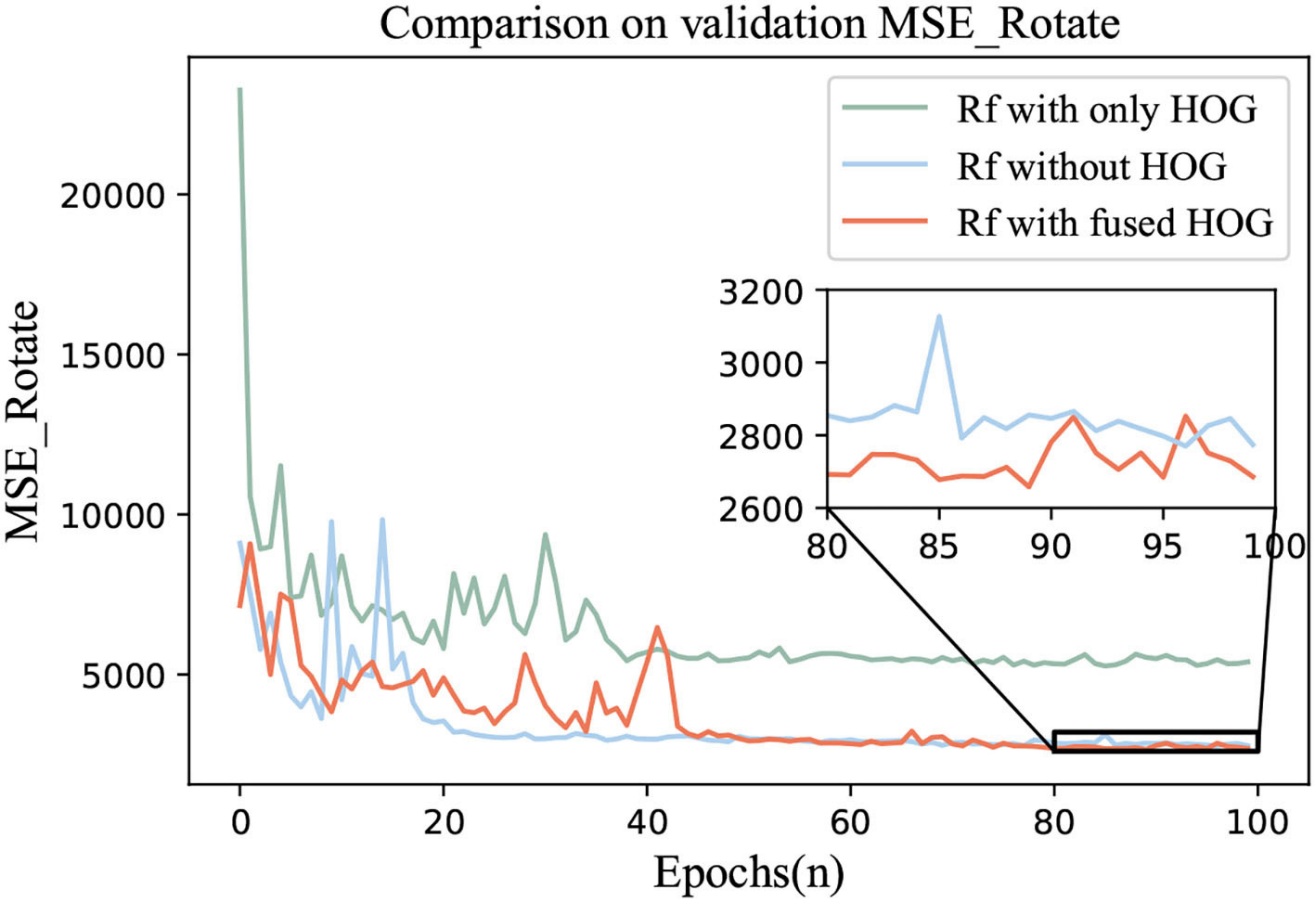
Comparison of MSE_Rotate values in ablation experiment.

The ablation results were shown in Figures 8, 9. The loss and MSE_Rotate of Rf with fused HOG feature could be lower and the prediction effect of rotation angle would be better.

### 4.6. Comparative experiment of networks

For comparison, as shown in Table 3, the error rate was used as the main measurement indicator. The minimum correct boundary angle difference in Katayama and Yamane (2017) was set at 22.5° and achieved an error rate within 10%. If within 11.25°, Rot-Minist was only 0.64%, which was significantly lower than 2.69% in Rodriguez et al. (2019) relying on the oriented wavelet feature. In our work, a boundary was limited to within 22.5°, 11.25°, and 3°, and the mean error rate on Rot-Minist was 0.59%, 0.64%, and 18.55%. Considering the complexity, the mean error rate on Circular-ImageNet was 27.67% with the angle error exceeding 22.5° as the boundary.

**Table 3:** Comparison of error rate (%) with other networks.

**Method**	**Correct** **boundary**	**Range**			

		**0**°	**90**°	**180**°	**360**°
Katayama and Yamane (2017)	22.5°	<10		\	
Rodriguez et al. (2019)	11.25°	2.69			\
Ours on Rot-Minist	22.5°	**0.59**			
	11.25°	**0.64**			
	3°	**18.55**			
Ours on Circular-ImageNet	22.5°	**27.67**			
	11.5°	**40.58**			

The bolded part means the effect of ETRNet.

### 4.7. Image righting

One application of blind detection of image rotation angle is to perform image righting. That is, we need to rotate the objects in the images to a normal angle.

**Figure 10:**
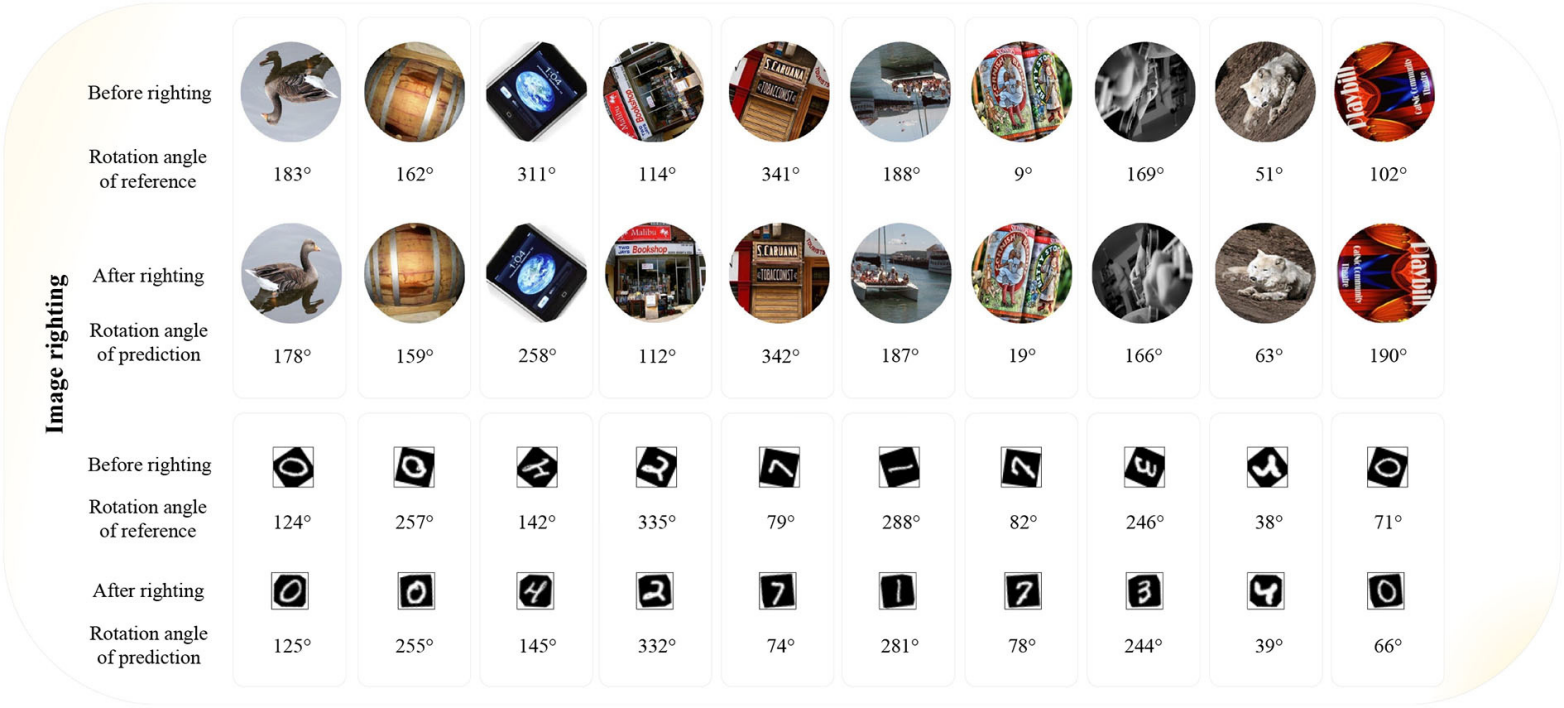
Examples of image righting.

Applying ETRNet to the detection of rotating images, the image can be corrected to the mode before rotation, that is, to complete the task of Image Righting. Relying on ETRNet, the effect of image righting can be shown in Figure 10.

## 5. Discussion

1. From 4.3, we can see that the Rotate Loss is indeed improved on the basis of Huber loss. It can converge to a lower range and obtain better MSE_Rotate results. It shows that our improvement does have a certain narrowing effect on the angle error.

2. As can be seen in Section 4.4, different regressors have different returns. When adopting transfer learning, it is necessary to comprehensively consider the impact of various strategies and choose better strategies as far as possible. For example, in our experiment, the Rf regressor can be regarded as a better regressor when the result of the validation set was the best and the result of the test set was relatively in the front position.

3. Ensemble transfer regression network may have more research potential than a single regressor. Through ETRNet, the MSE_Rotate decreased by 15–30%.

4. Ablation experiments also show that with the help of HOG, we can better control the prediction error of the rotation angle, and the MSE_Rotate can reach a lower level, which can be seen in the enlarged figure in Figure 9.

5. Compared with other networks, our strategy is also more advantageous and can be easily competent for blind detection of small datasets. The range of angles we can predict is larger and the error rate can be lower. However, in the case of large datasets with complex changes, our error rate still needs further control.

6. There is great space for the application of image righting in the real world. However, the experimental results also show that we can not guarantee very accurate rotation angle blind detection of a single image in complex scenes. For example, the third predicted angle error in Figure 10 was very large. However, by observing the image after righting, we can see that the effect of righting was basically in line with expectations.

## 6. Conclusion

In this paper, the ETRNet with fused HOG was used to realize the blind detection of the rotation angle of the circular image in the range of 0°–360°. Through the comparative experiments between different loss functions under the same network structure, it is verified that the proposed Rotate Loss has better convergence for ETRNet. Through the comparative experiment of designing multiple regressors, ETRNet was voted as the better regressor. As for the MSE_Rotate, Re could be as low as 28.79 on the training set of Circular-ImageNet and 2,686.09 on the validation set. The MSE_Rotate, MSE, MAE, and RMSE on the test set were 1,702.4325, 0.0263, 0.0881, and 0.1621, respectively. Based on the four better regressors, ETRNet achieved a good performance. We also tried to solve the application task of image righting and made some progress.

In future research, we can further optimize based on the network design in this paper, adopt a feature extractor with stronger performance, propose a regressor with better generalization, and improve the loss function to adapt to the rotation image angle detection in a variety of scenes. But it also means that the network may be more complex and have more parameters. Therefore, how to optimize the algorithm and improve the error rate may be a difficult problem.

## Data availability statement

The raw data supporting the conclusions of this article will be made available by the authors, without undue reservation.

## Ethics statement

Written informed consent was not obtained from the individual(s) for the publication of any potentially identifiable images or data included in this article.

## Author contributions

WD: experiments, data processing, writing the original draft, and funding acquisition. JZ: supervised the analysis and guided experiments. YZ and LG: research methods, and writing the original draft. XZ: performed the writing review. All authors have contributed to the paper and approved the submitted version.
